# SafeCrowdNav: safety evaluation of robot crowd navigation in complex scenes

**DOI:** 10.3389/fnbot.2023.1276519

**Published:** 2023-10-12

**Authors:** Jing Xu, Wanruo Zhang, Jialun Cai, Hong Liu

**Affiliations:** ^1^Key Laboratory of Machine Perception, Shenzhen Graduate School, Peking University, Shenzhen, China; ^2^School of Computer Science and Technology, Xidian University, Xi'an, China

**Keywords:** mobile robot, human-aware navigation, reinforcement learning, security assessment, collision avoidance

## Abstract

Navigating safely and efficiently in dense crowds remains a challenging problem for mobile robots. The interaction mechanisms involved in collision avoidance require robots to exhibit active and foresighted behaviors while understanding the crowd dynamics. Deep reinforcement learning methods have shown superior performance compared to model-based approaches. However, existing methods lack an intuitive and quantitative safety evaluation for agents, and they may potentially trap agents in local optima during training, hindering their ability to learn optimal strategies. In addition, sparse reward problems further compound these limitations. To address these challenges, we propose SafeCrowdNav, a comprehensive crowd navigation algorithm that emphasizes obstacle avoidance in complex environments. Our approach incorporates a safety evaluation function to quantitatively assess the current safety score and an intrinsic exploration reward to balance exploration and exploitation based on scene constraints. By combining prioritized experience replay and hindsight experience replay techniques, our model effectively learns the optimal navigation policy in crowded environments. Experimental outcomes reveal that our approach enables robots to improve crowd comprehension during navigation, resulting in reduced collision probabilities and shorter navigation times compared to state-of-the-art algorithms. Our code is available at https://github.com/Janet-xujing-1216/SafeCrowdNav.

## 1. Introduction

Mobile robots have been extensively studied and widely applied in recent decades as an essential branch of robotics research. They can accomplish tasks that are difficult or impossible for humans, reduce the workload of human workers, and improve people's quality of life. Our daily lives increasingly depend on mobile robots, which share living and social spaces with humans and interact with them to varying degrees. The crucial factor determining the successful autonomous movement of mobile robots across diverse environments is their possession of adaptable and autonomous navigation capabilities.

The key to achieving efficient autonomous navigation of mobile robots in various environments lies in key elements such as safety, autonomy, effectiveness, and user-friendliness. Among these, obstacle avoidance (Duguleana and Mogan, 2016; Pandey et al., 2017), serving as a primary means to ensure safety, poses a challenging research problem in robot navigation. It has been studied for decades and finds applications in critical real-world scenarios such as autonomous driving (Kästner et al., 2021) and cargo logistics. For instance, in the context of mobile robots, scenarios like autonomous navigation within unmanned supermarkets or warehouses, where robots navigate among shoppers or workers while avoiding obstacles, have garnered significant attention. At the same time, the operating environments for mobile robots have become increasingly complex, with various static and dynamic obstacles coexisting, including obstacles such as barriers, pedestrians, vehicles, or other robots. These scenarios add a layer of complexity, as robots must safely maneuver in dynamic environments alongside pedestrians and other obstacles, showcasing the versatility and practicality of mobile robotics. While classical planning methods (Cai et al., 2023) can effectively handle static environments, reliable obstacle avoidance in dynamic environments remains a significant challenge. Safe and reliable navigation in these highly dynamic environments is still a crucial challenge.

**Figure 1:**
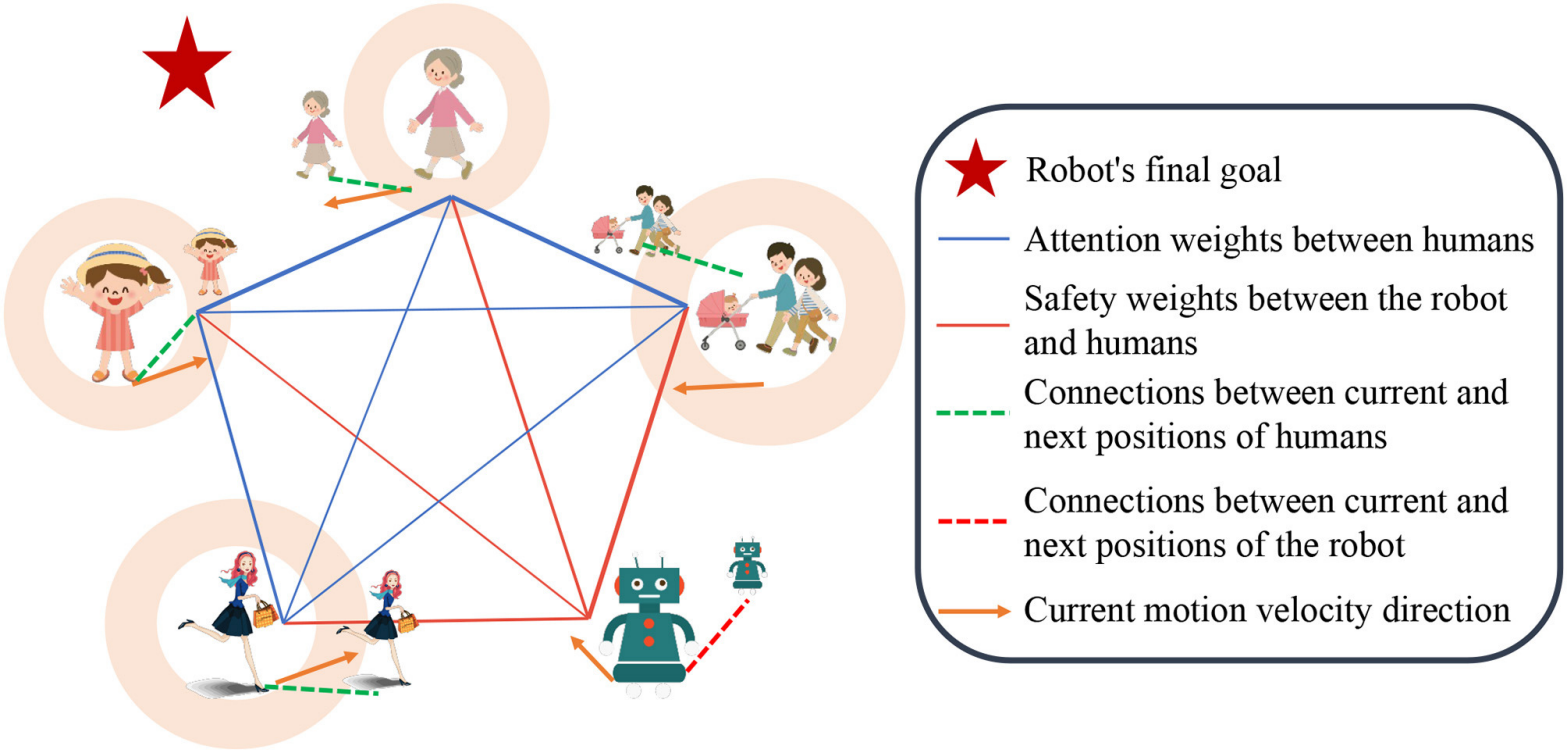
Illustration of our work: the robot utilizes heterogeneous attention weights and safety evaluation scores obtained from observations to selectively aggregate pedestrian information, enabling more anticipatory decision-making.

The illustration of our work is showing in Figure 1 and the paper presents the following key contributions:

- We design a novel framework called SafeCrowdNav, which integrates hindsight experience replay and prioritized experience replay to address the challenge of sparse-reward navigation.
- We firstly propose novel safety evaluation reward functions to estimate the safety weights of the robot in its current state, enabling more accurate obstacle avoidance during the navigation process.
- We firstly propose a novel intrinsic exploration reward function with visited count state that helps the robot avoid getting stuck in place and reduces unnatural robot behavior.

## 2. Related works

### 2.1. React-based collision avoidance

Over the past decade, extensive research has focused on robotic navigation in dynamic obstacle environments within the field of robotics. Numerous works have been dedicated to classical navigation techniques, with the earliest attempts being reactive rules-based methods, such as Optimal Reciprocal Collision Avoidance (ORCA) (Van den Berg et al., 2008), Reciprocal Velocity Obstacle (RVO) (Van Den Berg et al., 2011), and Social Force (SF) (Helbing and Molnar, 1995). These methods employ one-step interaction rules to determine the robot's optimal actions. However, despite considering interactions among agents, ORCA and SF simplify the crowd behavior model, leading to limitations such as shortsightedness, lack of safety, and unnatural movement patterns.

### 2.2. Trajectory-based collision avoidance

As a result, researchers have started exploring trajectory-based methods (Kothari et al., 2021) and considered visual-inertial initialization (Huang et al., 2021; Liu et al., 2022) to address crowd avoidance problems. Nevertheless, trajectory-based approaches suffer from high computational costs, inability to perform real-time updates in the presence of increasing crowd sizes and difficulties in finding safe paths (Trautman and Krause, 2010; Alahi et al., 2016; Sathyamoorthy et al., 2020). These limitations restrict the application and effectiveness of these methods in large-scale crowd scenarios.

### 2.3. Learning-based collision avoidance

To overcome the above challenges, recent research has modeled the crowd navigation problem as a Markov Decision Process (MDP) and introduced deep reinforcement learning called Collision Avoidance with Deep Reinforcement Learning (CADRL). Chen et al. (2019) propose the Socially Attentive Reinforcement Learning (SARL), which combines human-robot interaction features with self-attention mechanisms to infer the relative importance of neighboring humans with respect to their future states. They also develop the simulation environment CrowdNav (Chen et al., 2019), which has been widely used for comparing CADRL approaches. In CrowdNav, the information regarding the agent's position, velocity, and radius is considered as input, and the robot responds accordingly based on this input. To address the computational cost associated with learning-based methods, Zhou et al. (2022) propose SG-D3QN, which utilizes graph convolutional networks to predict social attention weights and refines coarse Q-values through online planning of potential future trajectories. The latest paper (Martinez-Baselga et al., 2023) claims to be the first work in this field that applies intrinsic rewards and has achieved the state-of-the-art performance.

### 2.4. Safety evaluation

However, reinforcement learning algorithms suffer from a fatal drawback: the need for trial and error exploration of the environment to learn optimal policies. In real-world settings, safety is a crucial concern, and trial and error that may cause harm to humans during the exploration process is unacceptable. Although current practices often train reinforcement learning agents in simulation environments with low safety risks, the complexity of transitioning from simulated environments to the real world poses a series of unacceptable safety issues (Ray et al., 2019). Therefore, safety evaluation should be a key focus area in reinforcement learning research. In this regard, this paper is dedicated to addressing safety concerns and proposes a robot crowd navigation system that enables the evaluation of an agent's safety performance.

## 3. Problem formulation

### 3.1. Crowd navigation modeling

The problem of crowd navigation for robots refers to guiding a robot to its target location in the shortest possible time while avoiding collisions with a variable number of intelligent agents behaving like a crowd in the environment. These agents can encompass various types of obstacles, and in this study, we utilize the CrowdNav simulation environment widely adopted in previous works (Chen et al., 2019, 2020; Everett et al., 2021).

The observable state of all agents *w* is represented by their positions *p* = [*p*_*x*_, *p*_*y*_], velocities *v* = [*v*_*x*_, *v*_*y*_], and radii *r*. The observable state indicates the information that other visible agents in the environment can perceive. Additionally, the state of the robot includes its preferred velocity (*v*_*p*_), heading angle (θ), and target coordinates (*g* = [*g*_*x*_, *g*_*y*_]). At a given time step *t*, the input joint state of the robot *s*^*t*^ is defined as:


(1) $$\begin{array}{l} \begin{array}{c} s^{t}=\left[w_{r}^{t},w_{h}\right]\\ w_{r}^{t}=\left[p_{x}^{t},p_{y}^{t},v_{x}^{t},v_{y}^{t},r^{t},g_{x}^{t},g_{y}^{t},v_{p}^{t},\theta^{t}\right]\\ w_{h}=\left[w_{1}^{t},w_{2}^{t},\ldots ,w_{n}^{t}\right]\\ w_{i}^{t}=\left[p_{x}^{i},p_{y}^{i},v_{x}^{i},v_{y}^{i},r\right],i>0, \end{array} \end{array}$$

where $w_{r}^{t}$ is the state of the robot *r*, $w_{i}^{t}$ is the state of human agent *i* and *w*_*h*_ is the collective state of all human agents.

### 3.2. Reinforcement learning based on the Q-value

In our work, the crowd navigation problem is formulated as a Markov Decision Process, and we adopt the double dueling deep Q-network as the fundamental method for solving this task. The objective is to estimate the optimal policy π^*^, which selects the optimal action *a*^*t*^ for state *s*^*t*^ at a specific time step *t*. The optimal policy maximizes the expected return, given by:


(2) $$\begin{array}{l} \pi^{*}\left(s^{t}\right)=\underset{a^{t}}{\mathrm{argmax}}\left(Q^{*}\left(s^{t},a^{t}\right)\right), \end{array}$$

where Q^*^ is the optimal action-value function, recursively defined with the Bellman equation as:


(3) $$\begin{array}{l} \begin{array}{c} Q^{*}\left(s^{t},a^{t}\right)=𝔼\left[r^{t}+\gamma^{\Delta t\cdot v_{p}}\underset{a^{t+1}}{\max}Q^{*}\left(s^{t+1},a^{t+1}\right)\right], \end{array} \end{array}$$

where *s*^*t*+1^ is the successor state and *r*^*t*^ is immediate reward. γ ∈ (0, 1) is the discount factor that balances the current and future rewards, normalized by the preferred velocity *v*_*p*_ and the time step size Δ*t*.

### 3.3. Reward shaping

While tackling the challenge of sparse reward tasks in crowd navigation without expert demonstrations, the most intuitive approach is to shape the reward function. However, previous works (Chen et al., 2017, 2019) have not given due attention to this aspect and instead applied sparse reward functions designed for non-communicative dyadic collision avoidance problems. In crowd navigation, such mismatched rewards can lead to poor training convergence (Chen et al., 2020). In contrast to existing reward functions (Chen et al., 2019; Zhou et al., 2022), which commonly rely solely on external or intrinsic rewards, our approach not only integrates and refines these two reward functions, but also introduces an additional safety evaluation function. We divide the overall reward *r*^*t*^ into three parts and innovate each: externally provided rewards $r_{ex}^{t}$, safety evaluation function $r_{safe}^{t}$, and intrinsic exploration rewards $r_{in}^{t}$, defined as follows:


(4) $$\begin{array}{l} r^{t}=r_{ex}^{t}+r_{safe}^{t}+r_{in}^{t}, \end{array}$$

where we first introduce innovations in the externally-provided reward function $r_{ex}^{t}$ offered by the environment to incentivize the robot to navigate toward the goal while avoiding collisions. Additionally, we introduced safety evaluation functions $r_{safe}^{t}$ and intrinsic rewards $r_{in}^{t}$ to encourage the robot to explore and exploit the environment while improving its safety and reliability.

## 4. Method

**Figure 2:**
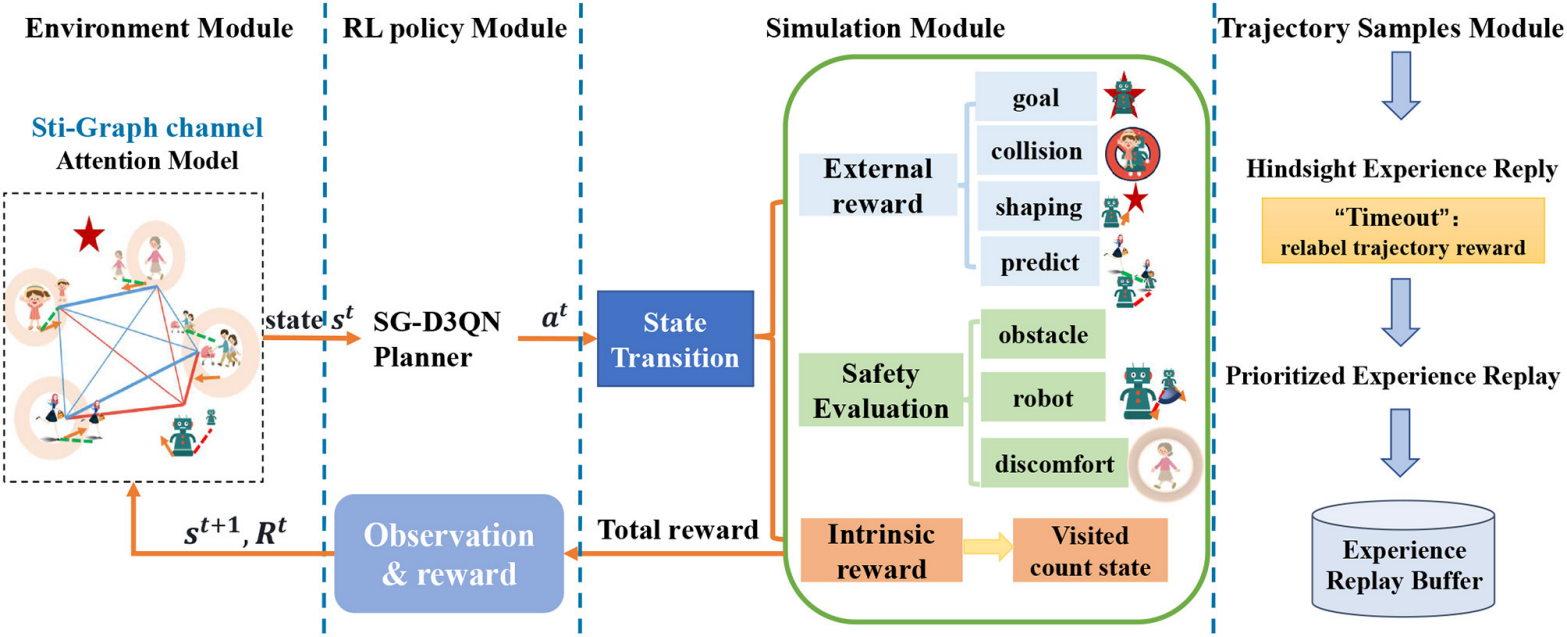
**Architecture of SafeCrowdNav**: **Environment Module**: Models the current environment information as a heterogeneous spatiotemporal graph. **RL Policy Module**: Implements an online planner based on SG-D3QN. Takes the state information as input and outputs the optimal action. **Simulation Module**: Divides the reward function in the reinforcement learning policy into three parts: intrinsic exploration reward, safety evaluation, and extrinsic reward. Optimizes the reward function. **Trajectory Sampling Module**: Combines hindsight experience replay and prioritized experience replay. Adjusts the reward for failed trajectories and performs experience importance sampling.

This paper focuses on the safety evaluation of crowd navigation using deep reinforcement learning. Building upon SG-D3QN (Zhou et al., 2022), we firstly model the social relationship graph (Liu et al., 2023), a heterogeneous spatio-temporal graph as input to the SG-D3QN planner to generate optimal actions. The simulated environment provides external reward function, safety evaluation scores and intrinsic exploration reward function based on the current state, which are then fed back to the reinforcement learning policy. The trajectory sampling process combines hindsight experience replay and prioritized experience replay to handle the data in the experience replay buffer. The overall framework of our algorithm is illustrated in Figure 2.

### 4.1. External reward function

We redesign the external reward function $r_{ex}^{t}$ offered by the environment, dividing it into $r_{goal}^{t}$, $r_{collision}^{t}$, $r_{shaping}^{t}$, $r_{pred}^{t}$ four components. $r_{goal}^{t}$ is used to reward the robot for reaching the goal, $r_{collision}^{t}$ penalizes collisions, $r_{shaping}^{t}$ guides the robot toward the goal, and $r_{pred}^{t}$ provides penalties for potential collisions in future time steps. Our external reward function is defined as follows:


(5) $$\begin{array}{l} r_{ex}^{t}=r_{goal}^{t}+r_{collision}^{t}+r_{shaping}^{t}+r_{pred}^{t}. \end{array}$$

The individual components $r_{goal}^{t}$, $r_{collision}^{t}$, $r_{shaping}^{t}$, $r_{pred}^{t}$ are defined as follows:


(6) $$\begin{array}{l} r_{\text{goal}}^{t}=\{\begin{array}{cc} r_{\text{arr}} & \text{if target is reached}\\ 0 & \text{otherwise} \end{array} \end{array}$$

(7) $$\begin{array}{l} r_{\text{collision}}^{t}=\{\begin{array}{cc} r_{\text{col}} & \text{if collision}\\ 0 & \text{otherwise} \end{array} \end{array}$$

(8) $$\begin{array}{l} r_{\text{shaping}}^{t}=w_{p}\cdot \left(\left\|p^{t-1}-p_{g}\|-\|p^{t}-p_{g}\right\|\right) \end{array}$$

(9) $$\begin{array}{l} r_{\text{pred}}^{t}=\underset{i=1,\ldots ,n}{\min}r_{\text{pred}}^{i,t}=\underset{i=1,\ldots ,n}{\min}\left[\underset{k=1,\ldots ,K}{\min}\left({𝟙}_{i}^{t+k}\frac{r_{\text{col}}}{2^{k}}\right)\right], \end{array}$$

where $r_{\text{shaping}}^{t}$ represents the difference between the distance from the endpoint at time *t* − 1 and *t*. *p*^*t*^ and *p*_*g*_ respectively represent the robot's position and the goal at time *t*, and *w*_*p*_ is a hyper-parameter. Prediction reward function $r_{\text{pred}}^{t}$ presents the maximum penalty for collisions occurring among *n* humans in future *K* time steps. ${𝟙}_{i}^{t+k}$ indicates whether the robot collides with the predicted position of the human *i* at time *t* + *k*. The role of 2^*k*^ is to assign different weights to collisions at different predicted time steps, with lower penalty weights given to collisions predicted farther into the future.

### 4.2. Safety evaluation function

The safety evaluation function $r_{safe}^{t}$ assesses the current safety level of the robot based on the surrounding environment information and adjusts the robot's behavior accordingly to guide it toward safer navigation. Specifically, if the safety evaluation function $r_{safe}^{t}$ provides a higher safety score, it indicates a lower risk and likelihood of collisions in the current environment, allowing the robot to choose a relatively higher speed to complete the navigation task more quickly. Conversely, if the safety evaluation function $r_{safe}^{t}$ provides a lower safety score, it indicates a higher risk and likelihood of collisions in the current environment, requiring the robot to lower its speed or even stop to avoid potential danger. The factors considered in the safety evaluation function include:

(1) Collision probability $r_{obstacle}^{t}$ between the robot and obstacles: It considers the movement speed and direction of obstacles, the distance between the robot and obstacles, and the obstacle type together. A global collision probability map is used here, where closer obstacles to the robot have a higher collision probability *p*_collision_.

(2) Robot's velocity $r_{robot}^{t}$: Ensuring smooth and natural motion is vital in dynamic and crowded settings, enhancing comfort and safety for passengers and bystanders. Abrupt velocity changes can cause discomfort and confusion among humans and destabilize navigation, leading to collisions. Thus, we quantify motion smoothness by assessing continuity in velocity changes, calculated from the cosine of the angle between current *v*^*t*^ and previous *v*^*t*−1^ robot actions.

(3) Safety distance $r_{discomfort}^{t}$ between obstacles and the robot: To ensure the safety and comfort of humans during robot navigation, we additionally impose a penalty when the distance between obstacles and the robot falls below the predefined safety threshold. Actually, collision probability $r_{obstacle}^{t}$ can partially achieve this goal, but only use it fail to discourage situations that may potentially cause discomfort to humans.

The composition of the safety score is as follows:


(10) $$\begin{array}{l} r_{safe}^{t}=r_{obstacle}^{t}+r_{robot}^{t}+r_{discomfort}^{t} \end{array}$$

(11) $$\begin{array}{l} r_{\text{obstacle}}^{t}=\beta \cdot p_{\text{collision}} \end{array}$$

(12) $$\begin{array}{l} r_{robot}^{t}=\alpha \cdot \frac{\vec{v^{t-1}}\cdot \vec{v^{t}}}{|\vec{v^{t-1}}||\vec{v^{t}|}} \end{array}$$

(13) $$\begin{array}{l} r_{discomfort}^{t}=\sum_{i=1}^{N}f\left(d_{i}^{t},d_{s}\right)\\ f(d_{i}^{t},d_{s})=\{\begin{array}{ll} d_{i}^{t}-d_{s} & \text{if }d_{i}^{t}<0.2\\ 0 & \text{else} \end{array}, \end{array}$$

where β is a hyper-parameter, *p*_collision_ is our collision probability and *v*^*t*^ represents the velocity of the robot at the current time step *t*. Discomfort reward function $r_{discomfort}^{t}$ encourages the robot to maintain a safe distance from all pedestrians, where *d*_*s*_ is the minimum safe distance that the robot needs to maintain with pedestrians at any time. In this paper, *d*_*s*_ is set to 0.2 m, $d_{i}^{t}$ represents the actual minimum distance between the robot and the *i*-th pedestrian within the time step.

Inspired by Wang et al. (2022), our collision probability *p*_collision_ is:


(14) $$\begin{array}{l} p_{\text{collision}}=\overset{i=1,\ldots ,n}{\underset{\left(x,y\right)\in \phi_{\text{human}}}{\sum }}g_{i}\left(x,y\right), \end{array}$$

where ϕ_human_ represents the range of human perception, determined by the velocities of the robot and humans and the unit of time. *g*_*i*_(*x, y*) denotes the collision probability of the robot relative to human *i*. “Arrive” refers to the distance between the agent and its target position being less than 0.1 m. At time *t*, *g*_*i*_(*x, y*) can be computed as follows:


(15) $$\begin{array}{l} g_{i}^{t}\left(x^{t},y^{t}\right)=\overset{N}{\underset{i=1}{\sum }}N\left(\delta_{x},x\right)\cdot N\left(\delta_{y},y\right)\cdot N\left(\delta_{\theta },\theta \right) \end{array}$$

(16) $$\begin{array}{l} N\left(\delta ,a\right)=\frac{\delta }{\sqrt{2\pi }}e^{-\frac{{\left(a^{t}-a_{i}^{o}\right)}^{2}}{2}} \end{array}$$

(17) $$\begin{array}{ll} \theta_{i}^{o}=\arctan & \left(\frac{v_{i}^{y}}{v_{i}^{x}}\right)\;\theta^{t}=\arctan\left(\frac{y^{t}-y_{i}^{o}}{x^{t}-x_{i}^{o}}\right), \end{array}$$

where *N* is the number of obstacles, and δ_*x*_, δ_*y*_, and δ_*z*_ are hyper-parameters representing variances. $\left(x_{i}^{o},y_{i}^{o}\right)$ represents the position of obstacle *i*, and $\theta_{i}^{o}$ denotes the heading angle of obstacle *i*. θ^*t*^ is the angle between the line from the robot's position (*x*^*t*^, *y*^*t*^) to the obstacle *i*s position $\left(x_{i}^{o},y_{i}^{o}\right)$ and the x-axis.

Finally, the safety scores are introduced to assess the safety of the current environment. Based on these scores, the robot's behavior is modified to navigate and avoid collisions with the crowd. This approach aims to reduce the risk of collision by providing real-time analysis and guidance in response to the assessed safety levels.

### 4.3. Intrinsic reward function

The intrinsic reward encourages the robot to explore new states or reduce the uncertainty of predicted action outcomes (Badia et al., 2020). In this work, the intrinsic reward incentivizes the agent to visit unknown or unpredictable states until they are adequately explored and exploited, particularly in the vicinity of humans and the goal. Incorporating intrinsic exploration is beneficial in this context. Our approach is based on the Intrinsic Curiosity Module (ICM) (Pathak et al., 2017).

First, the states *s* and next states *s*_*t*+1_ are encoded as inputs to the feature encoder network ϕ, resulting in feature representations in the feature space ϕ(*s*_*t*_) and ϕ(*s*_*t*+1_). This step aims to transform the agent-level states into state representations defined by feature vectors as outputs of the feature encoder network. Then, the states in the feature space are used to predict the actions taken, denoted as $\hat{a_{t}}$. Simultaneously, the actual actions *a* and the feature space states ϕ(*s*_*t*_) are used to predict the next states in the feature space $\hat{\phi }\left(s_{t+1}\right)$. We adopt the same feature encoder network as (Martinez-Baselga et al., 2023), and the intrinsic reward is calculated as the mean squared error (MSE) between ϕ(*s*_*t*+1_) and $\hat{\phi }\left(s_{t+1}\right)$, where higher MSE indicates that the agent is accessing unknown or unpredictable states.

To tackle the challenge of inefficient navigation resulting from excessive exploration, such as repetitive behavior within the same area, we have incorporated a state visitation record mechanism. This enhancement optimizes the exploration strategy and effectively curbs trajectory loops. The intrinsic reward *r*_*in*_ is formulated as follows:


(18) $$\begin{array}{l} \begin{array}{c} r_{\text{in}}=\mu \frac{\mathrm{MSE}\left(\phi \left(s_{t+1}\right),\hat{\phi }\left(s_{t+1}\right)\right)}{\sqrt{C\left(s_{t+1}\right)}}, \end{array} \end{array}$$

where μ is a hyper-parameter and *C*(*s*_*t*_) represents the visited count of states at time step *t*, indicating the number of times the robot has observed state *s*_*t*_. The visited count is used to drive the robot out of already visited areas to avoid trajectory loops in the same region. The visited count state is computed on a per-episode basis, *C*_*ep*_(*s*_*t*_) = *C*(*s*_*t*_).

### 4.4. Experience replay

Traditional experience replay algorithms only store the experiences generated by the interaction between the agent and the environment (i.e., state, action, reward, and next state) and randomly sample them for training the agent. However, these approaches overlook valuable information, such as the agent's erroneous decisions and the significance of experiences. Errors in decision-making provide valuable learning opportunities for agents to improve their future actions, while the significance of experiences helps prioritize the replay of important events, allowing agents to learn more efficiently from crucial interactions. Therefore, we propose combining the prioritized experience replay and hindsight experience replay algorithms.

The key advantage of Prioritized Experience Replay (PER) (Schaul et al., 2015) lies in its ability to prioritize and sample important experiences, thereby enabling more effective utilization of the agent's training data. PER introduces a priority queue that efficiently sorts experiences based on their importance for training the agent, giving higher priority to experiences that are more beneficial for training. The sampling probability, denoted as *P*(*i*), is monotonic with respect to the priority of the transition, ensuring a non-zero probability even for transitions with the lowest priority. In our approach, we adopt the rank-based prioritization sampling method *p*(*i*) in order to enhance robustness and reduce sensitivity to outliers:


(19) $$\begin{array}{l} P\left(i\right)=\frac{p_{i}^{\alpha }}{\sum_{k}p_{k}^{\alpha }} \end{array}$$

(20) $$\begin{array}{l} p_{i}=\frac{1}{\mathrm{rank}\left(i\right)}, \end{array}$$

where α is a hyper-parameter that determines the degree of prioritization in the sampling and controls the exponentiation of the priorities *p*_*i*_ in the calculation of the sampling probabilities *P*(*i*). Higher values of α emphasize experiences with higher priorities, enabling a more focused exploration of important experiences during replay.

Hindsight Experience Replay (HER) (Andrychowicz et al., 2017) addresses the specific case of failed experiences. While traditional experience replay algorithms overlook valuable information gained from failed experiences, HER can transform failed experiences into successful ones and add them to the experience replay buffer, thus effectively leveraging the knowledge from unsuccessful attempts. The key idea is to treat the final state as an additional goal, allowing the agent to learn useful information from failed simulated trajectories as if the agent had intended to reach that state from the beginning.

We present enhancements to the proposed algorithm (Li et al., 2021) tailored to suit our specific task better. Specifically, when a collision occurs or the agent reaches the goal in each episode, we store the trajectory in the experience replay buffer. If the agent's final state exceeds the global time limit (“Timeout”) without causing discomfort to humans (i.e., the shortest distance is less than the safety distance), we relabel the final state as reaching the goal and assign the last reward as half of the success reward. The modified trajectory is then stored in the replay buffer. The HER method is a straightforward approach without complex reward engineering, contributing to improved sample efficiency in reinforcement learning. The details of the HER algorithm are outlined in Algorithm 1.

**Algorithm 1:**
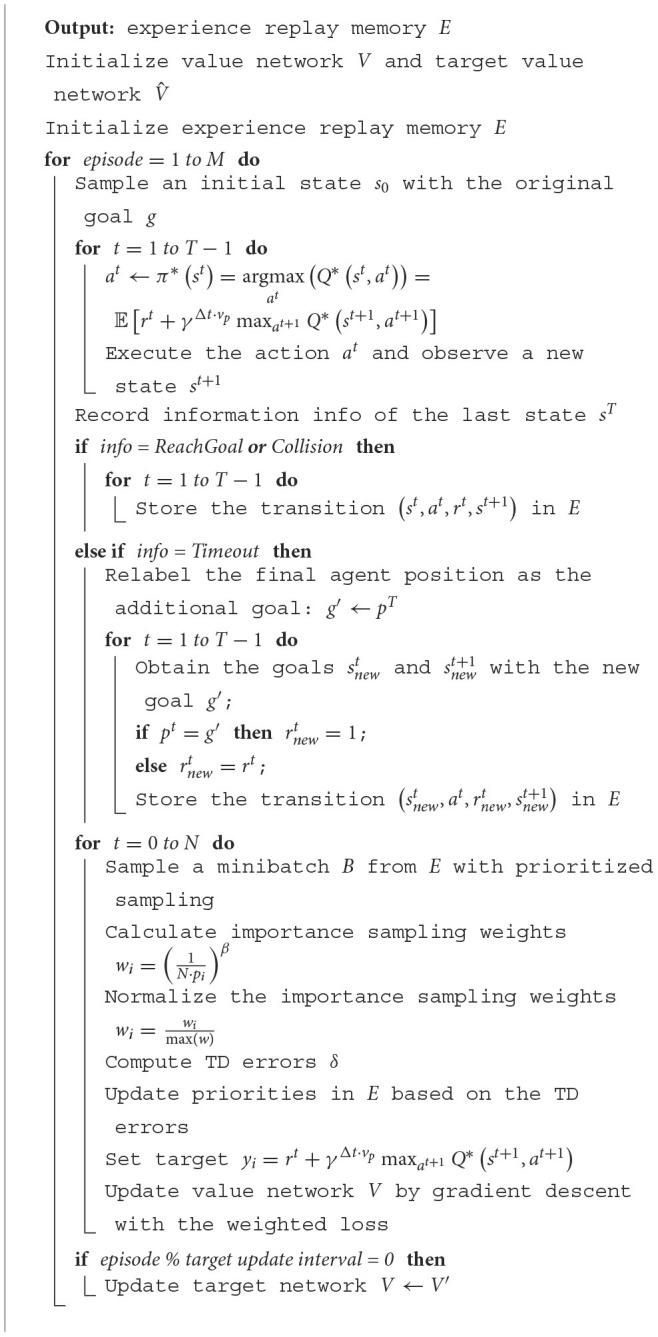
D3QN with HER and PER algorithm.

## 5. Experiments

### 5.1. Implementation details

This paper uses Open-Gym to create a simulation environment for modeling crowd behavior and conducting path planning. Specifically, we build upon the commonly used CrowdNav simulation environment (Chen et al., 2019), which simulates crowd behavior in indoor scenarios. It incorporates factors such as crowd density and movement directions, enabling us to better study crowd behavior and path planning problems, as well as facilitating algorithm comparison.

Within each scene of the CrowdNav environment, we set up five dynamic obstacles within a circular area, requiring them to pass through the center of the circle. In more complex scenarios, we add five randomly placed individuals who must traverse the room. They navigate using the ORCA (Van den Berg et al., 2008) algorithm to avoid collisions with each other. The robot is invisible to them, meaning pedestrians in the simulation will never yield to it. This necessitates the robot to have a more proactive and anticipatory collision avoidance strategy, requiring it to execute complete obstacle avoidance maneuvers. When one person reaches a specified goal, another goal is randomly assigned to prevent them from stopping.

A total of 10,000 randomly generated episodes (agents with random positions and trajectories) are trained in this study. Each algorithm starts with the same randomly initialized weights to ensure a fair comparison. The training hardware is a computer with an AMD Ryzen 5600X CPU and an Nvidia GeForce RTX 3090 GPU, which can simultaneously train four tasks overall in three days.

### 5.2. Quantitative evaluation

The baseline of our approach is intrinsic-SGD3QN (Martinez-Baselga et al., 2023), which innovatively introduces intrinsic exploration rewards on top of the related work SG-D3QN (Zhou et al., 2022). Building upon the CrowdNav simulation environment, this work introduces the innovative concept of intrinsic exploration reward. In addition, we incorporate prioritized experience replay, hindsight experience replay, the intrinsic curiosity module with visit count of states, and safety evaluation for exploration. We explore different hyper-parameters and select the best ones in each case. To validate and compare these methods, each method is tested in 10,000 randomly generated episodes in circular scenes. Table 1 compares state-of-the-art methods and our approach, highlighting success rate, collision rate, navigation time, and average return as performance metrics.

**Table 1:** Quantitative results: “Success:” the rate of the robot reaching its goal without a collision. “Collision:” the rate of the robot colliding with other humans. “Nav. Time:” the robot's navigation time to reach its goal in seconds. “Avg. Return:” discounted cumulative reward in a navigation task.

**Method**	***Successs*↑**	***Collision*↓**	***Nav. Time*↓**	***Avg. Return*↑**
OCRA (Van den Berg et al., 2008)	0.736	0.252	13.865	0.3234
AEMCARL (Wang et al., 2022)	0.920	0.045	12.859	0.5392
Intrinsic-SGD3QN (Martinez-Baselga et al., 2023)	0.966	0.034	**9.793**	0.6964
Hindsight & prioritized experience reply (ours)	0.948	0.052	11.753	0.6194
Intrinsic-Ntimes (ours)	0.977	0.023	10.036	0.7028
Experience reply & intrinsic-Ntimes (ours)	0.980	0.019	10.282	0.6953
**SafeCrowdNav(ours)**	**0.986**	**0.014**	9.984	**0.7070**

Bold values indicate the best performance of four metric.

The results in the table indicate that our method SafeCrowdNav significantly improves the original results and outperforms other methods. The utilization of prioritized experience replay and hindsight experience replay enhances the efficiency of the agent in utilizing past experiences. Our approach's additional safety evaluation function achieves a success rate of 98.6%, which is a 2% improvement compared to the baseline. Our method also demonstrates the ability to find near-optimal solutions quickly and reduces collision probability by 2%, thereby improving the robustness of navigation.

### 5.3. Qualitative evaluation

**Figure 3:**
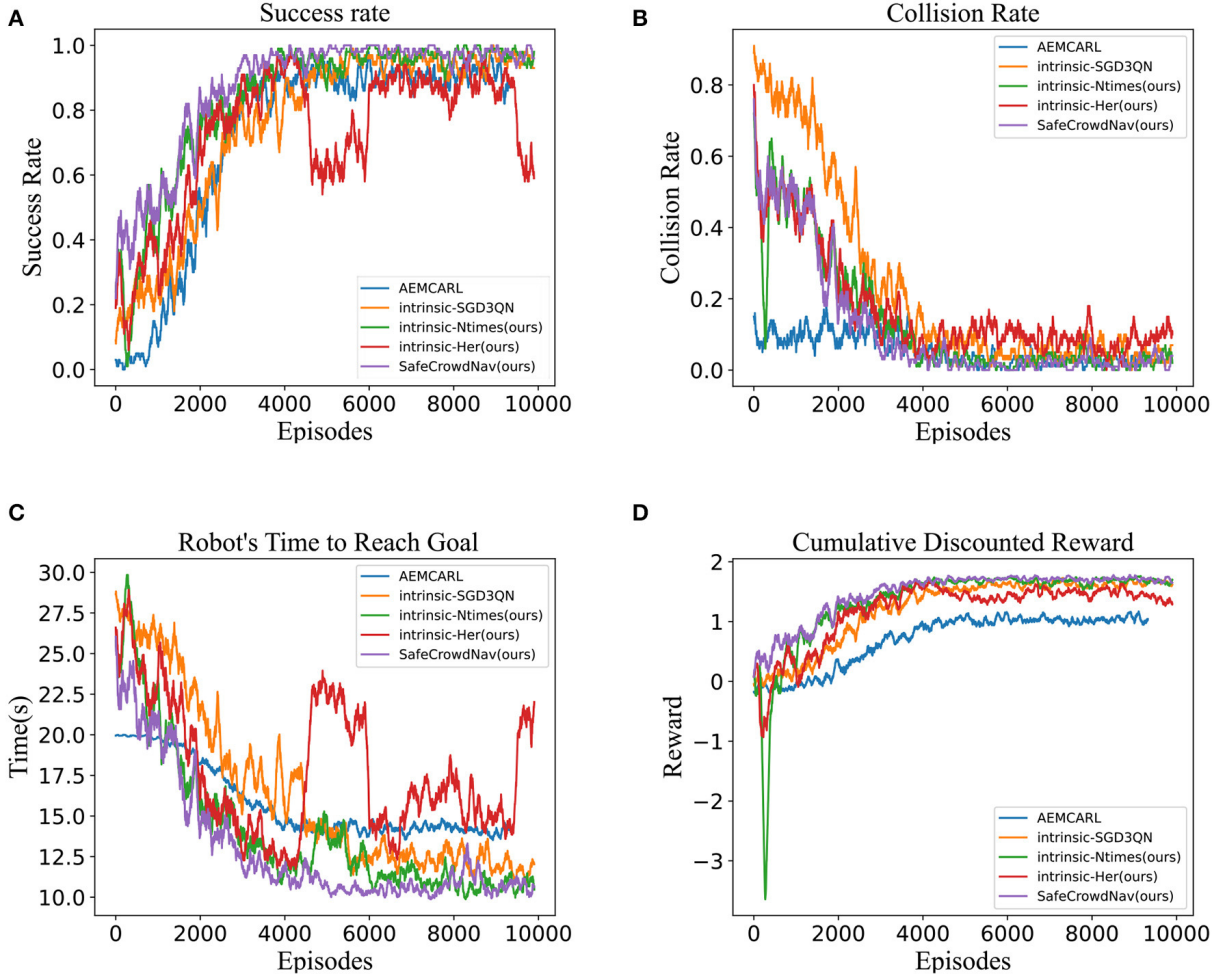
Navigation performance about success rate, collision rate, time to reach the goal, and cumulative discounted reward over 10,000 training episodes. **(A)** Success rate. **(B)** Collision rate. **(C)** Time to reach the goal. **(D)** Cumulative discounted reward.

In the simple scenario, the training curve is depicted in Figure 3. The metrics of our method SafeCrowdNav are plotted in orange, AEMCARL (Wang et al., 2022) in blue, Intrinsic-SGD3QN (Martinez-Baselga et al., 2023) in purple and the remaining colors are the metrics of our ablation experiments. It obvious reveals that our method outperforms Intrinsic-SGD3QN (Martinez-Baselga et al., 2023) on four metrics. At the beginning of training, with a randomly initialized model, it is challenging for the agent to accomplish the crowd navigation task, and most of the termination states result in “Timeout” or “Collision.” As training progresses, the robot quickly learns to maintain a safe distance from pedestrians. It gradually comprehends the crowd's behavior and plans its path based on its predictions of pedestrian trajectories. The robot's performance becomes relatively stable toward the end of the training.

**Figure 4:**
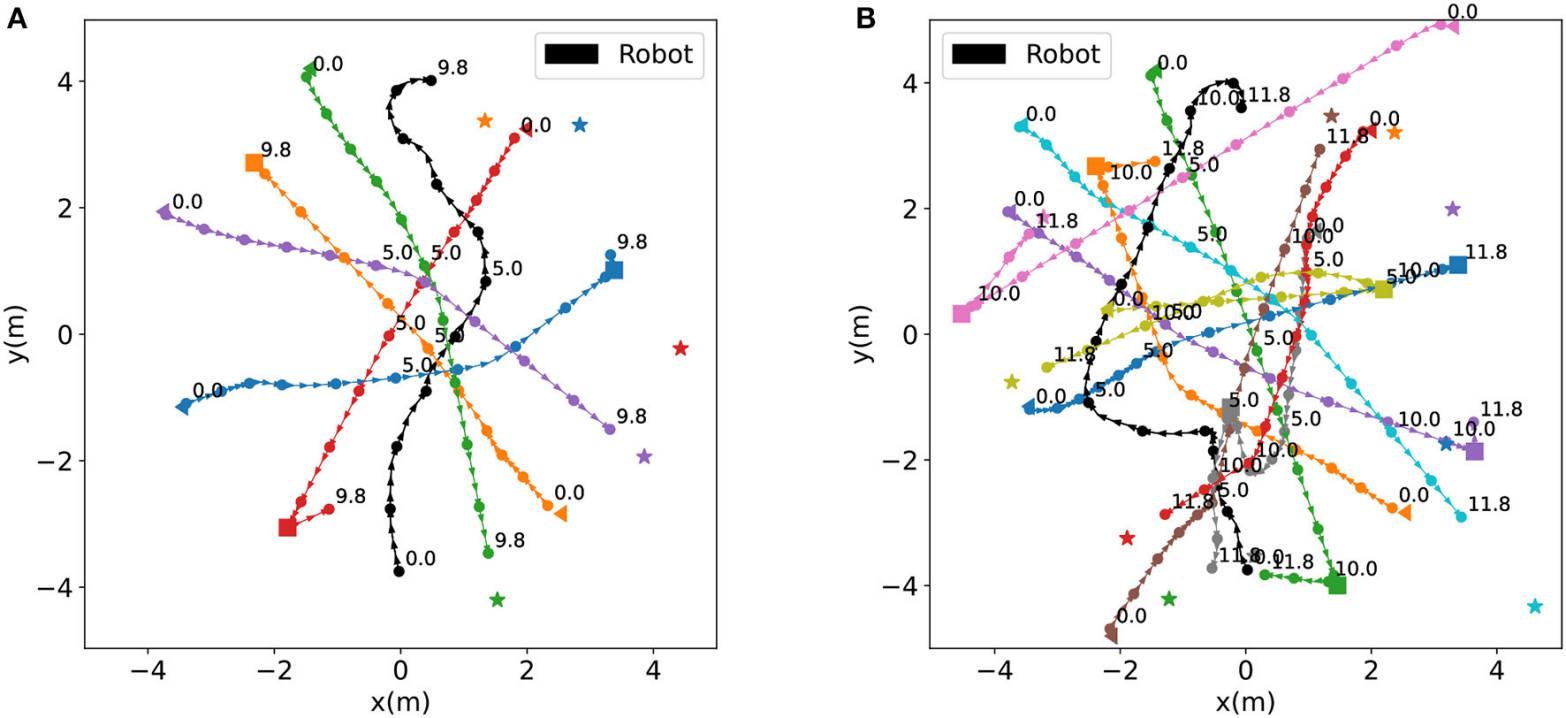
Trajectory maps for a simple and a complex scene. In these maps, the circles represent agents, with the black circle representing the robot and other colors representing pedestrians. The numbers near the circles indicate the corresponding time steps. The time interval between two consecutive circles is 1.0 seconds. The maps mark humans' starting positions, turning points, and final goal positions with triangles, squares, and pentagrams, respectively. **(A)** Trajectories in a simple scenario. **(B)** Trajectories in a complex scenario.

Through learning-based strategies, the robot is able to reach the target location safely and quickly in both simple and complex scenarios, as depicted in Figures 4A, B. In the complex scenario, the robot needs to pay more attention to avoid pedestrians, resulting in rougher trajectories, and longer navigation times. In both simple and complex scenarios, the robot exhibits proactive, and anticipatory collision avoidance behavior. The robot can recognize and avoid interaction centers where pedestrians approach each other. For instance, in the simple scenario, the robot suddenly turns right at around 4.0 seconds to avoid a potential encirclement at 5.0 seconds. Additionally, in complex scenarios, even when the robot is surrounded by pedestrians, it possesses the ability to safely escape the environment. In this particular instance, the encirclement by three pedestrians starts at 1.0 seconds and lasts for approximately 3.0 seconds.

**Figure 5:**
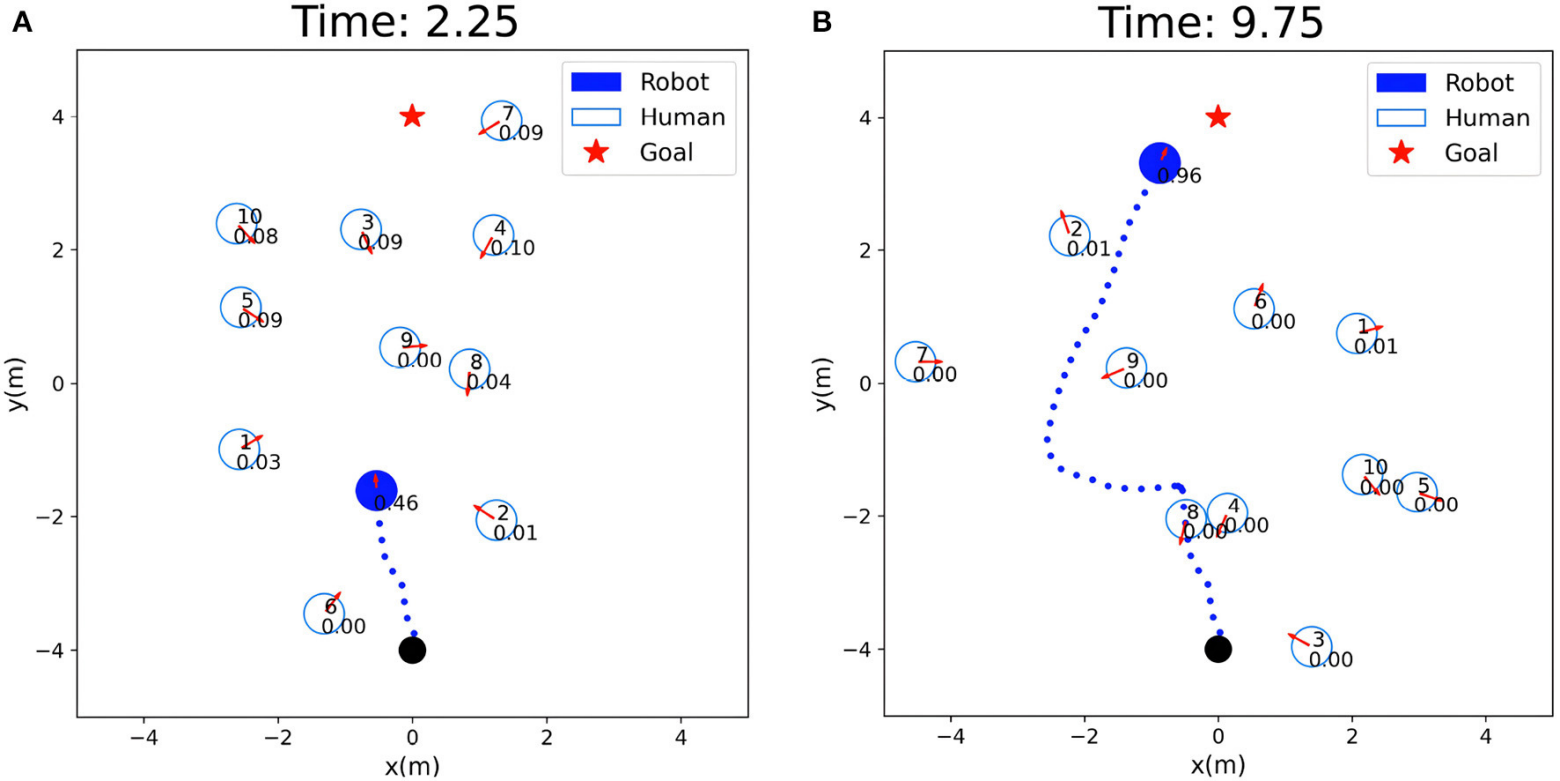
Visualization of safety evaluation scores: the solid circle represent the robot, the hollow circles represent humans, and the numbers inside the circles indicate the safety evaluation scores of the robot. **(A)** Low safety evaluation score: 0.46. **(B)** High safety evaluation score: 0.96.

The safety evaluation in the tested crowd scenarios is shown in Figure 5, where the real-time safety evaluation score of the robot for the current scene is dynamically displayed. A higher score indicates better safety in the current situation, guiding the robot to navigate faster, while a lower score indicates higher risk, prompting the robot to reduce speed and pay more attention to pedestrians moving toward it or potentially interacting with it. In Figure 5A, the robot's score is 0.46, indicating a lower score due to multiple pedestrians and a complex environment. The lower safety evaluation score guides the robot to reduce speed and allocate different attention weights to surrounding pedestrians, prioritizing obstacle avoidance. In Figure 5B, the robot's score is 0.96, indicating fewer pedestrians in the vicinity and guiding the robot to accelerate its movement, focusing more on navigation tasks. The setting of the safety evaluation score also helps the robot better balance navigation tasks and obstacle avoidance behavior.

## 6. Conclusion

This paper aims to address safety, autonomy, effectiveness, and user-friendliness in evaluating intelligent robot behaviors. We propose SafeCrowdNav, an innovative approach based on Deep Reinforcement Learning to enhance navigation in crowded environments. Our approach includes heterogeneous spatial-temporal maps for comprehensive environmental representation. We introduce a novel safety evaluation framework based on environment complexity and task difficulty. Additionally, we enhance the intrinsic reward by introducing constraints based on previously encountered scenes, effectively avoiding repetitive and inefficient exploration behavior by the agent. To facilitate efficient and safe navigation in dense crowds, we also integrate prioritized and hindsight experience replay techniques. Extensive evaluations in the CrowdNav simulator demonstrate that SafeCrowdNav achieves shorter trajectories and higher success rates compared to state-of-the-art algorithms.

However, future works still have many shortcomings to overcome. This includes the need for real-world scenario datasets to enhance performance in real environments, incorporating more realistic human reactions, and exploring the generalization performance from virtual to real-world scenarios. Adjusting the robot's shape based on real-world conditions and conducting real-world observations will provide valuable insights.

## Data availability statement

The original contributions presented in the study are included in the article/supplementary material, further inquiries can be directed to the corresponding author.

## Author contributions

JX: Conceptualization, Formal analysis, Investigation, Methodology, Software, Writing—original draft, Writing—review and editing. WZ: Conceptualization, Writing—original draft. JC: Writing—review and editing, Investigation. HL: Conceptualization, Funding acquisition, Resources, Supervision, Writing—review and editing.
